# Cilia, Wnt signaling, and the cytoskeleton

**DOI:** 10.1186/2046-2530-1-7

**Published:** 2012-05-02

**Authors:** Helen L May-Simera, Matthew W Kelley

**Affiliations:** 1 Laboratory of Cochlear Development, National Institute on Deafness and Other Communication Disorders, NIH, 35 Convent Drive. Bethesda, MD 20892, USA

**Keywords:** Cilia, basal body, Wnt signaling, PCP, β-catenin, microtubules, actin, cytoskeleton, kinesins, GTPases

## Abstract

Primary cilia have recently been highlighted as key regulators in development and disease. This review focuses on current work demonstrating the broad role of cilia-related proteins in developmental signaling systems. Of particular consideration is the importance of the basal body region, located at the base of the cilium, in its role as a focal point for many signaling pathways and as a microtubule organizing center. As the cilium is effectively a microtubular extension of the cytoskeleton, investigating connections between the cilium and the cytoskeleton provides greater insight into signaling and cell function. Of the many signaling pathways associated with primary cilia, the most extensively studied in association with the cytoskeleton and cytoskeletal rearrangements are both canonical and non-canonical Wnt pathways. One of the key concepts currently emerging is a possible additional role for the traditionally 'cilia-related' proteins in other aspects of cellular processes. In many cases, disruption of such processes manifests at the level of the cilium. While the involvement of cilia and cilia-related proteins in signaling pathways is currently being unraveled, there is a growing body of evidence to support the notion that ciliary proteins are required not only for regulation of Wnt signaling, but also as downstream effectors of Wnt signaling. This review summarizes recent advances in our understanding of the involvement of cilia and basal body proteins in Wnt signaling pathways.

## Introduction

The primary cilium had long been ignored by biologists and was considered to be a vestigial remnant of evolution, similar to the human appendix. However, a growing number of human disorders are being attributed to dysfunctions in cilia or cilia-related proteins [1], leading to the term ciliopathies to describe these disorders. Ciliopathies display high degrees of clinical variability, genetic heterogeneity, and phenotypic overlap [2], complicating their discovery and diagnosis. But, in recent years an explosion of data has linked the cilium to several crucial cellular processes and various signaling pathways. Initially cilia were classified as either motile or primary cilia based on their internal structure. Motile cilia were thought to be comprised exclusively of nine outer microtubule doublets with a central pair of microtubules (9+2). In contrast, primary cilia (also referred to as sensory cilia) lack the central microtubule doublets (9+0). Motile cilia are known to be required for mucus clearance, cerebrospinal fluid flow, sperm motility, and leftward flow at the embryonic node, among other functions [3], and defects in this motility are known to be associated with diseases such as Kartagener syndrome (primary ciliary dyskinesia). Primary cilia, in contrast, while expressed on virtually every cell, were considered to be non-functional, evolutionary remnants.

More recently, the primary ciliary membrane has been shown to be rich in various channels and receptors [4,5], suggesting that the cilium may serve as the signaling antenna for the cell. In this capacity, cilia might sense developmental morphogens, growth factors, hormones, odorants, and other extracellular signals. Moreover, the cilia-associated basal body complex appears to serve as a gatekeeper for the regulation of downstream intracellular signaling events that are initiated as a result of ciliary receptor activation. As the cilium has no protein synthetic machinery of its own, all the components required for both the formation and function of the cilium have to be transported from the cytoplasm of the cell. This process requires intraflagellar transport (IFT) [6], defects in which have significant impact on a wide range of biological processes. Many proteins associated with cilia, basal body, or IFT function are also thought to regulate cytoskeletal assembly and/or intracellular trafficking. Our understanding of how mutations in cilia-associated proteins lead to disruptions in cilia function and subsequent cellular pathology remains limited, but valuable insights are beginning to emerge [5-7].

This review will highlight our current understanding of the cilium and basal body as one of the microtubule organizing centers of the cell and how they function as a critical signaling center. The cilium has been implicated in the regulation of a growing number of signaling pathways, and while much recent research has focused on sonic hedgehog (Shh)-signaling, this review will more closely examine the role of the cilium and basal body in regard to Wnt signaling, an as yet less explored avenue of research. To begin, we will take a closer look at the cilium and related proteins, as well as, the possible roles of those proteins beyond the cilium. Then, the role of cilia and basal bodies in both canonical and non-canonical Wnt signaling will be examined; focusing more specifically on their association with cytoskeletal regulation.

## The Cilium - Structure and Function

**Figure 1:**
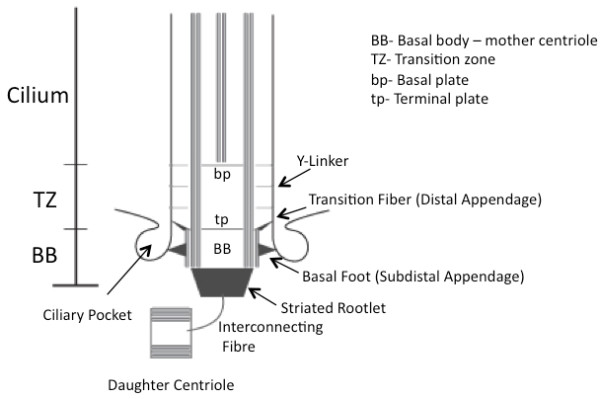
**Ultrastructural depiction of the base of a generic mammalian cilium**. The basal body (BB), derived from the mother centriole, nucleates the microtubule axoneme of the cilium. The basal body ends at the terminal plate (tp) and the transition fibers. The transition fibers are electron dense fibers at the base of the cilium connecting the ciliary axoneme to the plasma membrane. The transition zone (TZ) is characterized by the presence of the ciliary necklace on the ciliary membrane (not depicted) and Y-linkers, the end of which correspond with the basal plate (bp). In cilia that contain central microtubules, these emanate from the basal plate. The transition fibers and transition zone encompass the so-called 'ciliary gate', which possibly regulates protein entrance and exit. The fibers act as docking sites for intraflagellar transport particles and their motors, and could form part of a pore complex similar to the nuclear pores. The daughter centriole, connected to the basal body via an interconnecting fiber, and striated rootlet are also depicted. The ciliary pocket is an invagination of specialized cell membrane at the base of the cilium likely to be important for regulation of cilia composition.

Cilia are microtubule-based organelles, extending from the plasma membrane and coupled to the cytoskeleton. They are anchored to the cell and emanate from the basal body, a barrel structure of nine triplet microtubules that elongate to form the microtubule doublets of the ciliary axoneme. The basal body is derived from the mother centriole, and is also considered a microtubule-organizing centre (MTOC). Centrioles are more than just the primary MTOC of a cell; they also serve as important regions for protein interactions, and as docking stations for proteins on the way to the cilium [8,9]. The region at the base of the cilium is highly complex. Distal to the basal body is the transition zone, which starts where the triplet microtubules end and extends to the basal plate, upon which the central pair of microtubules (in the case of a motile cilium) are nucleated (Figure 1).

In eukaryotic cilia the transition zone is composed of several distinct structures including transitional fibers, Y-linkers, and the ciliary necklace (on the cilia membrane), all flanked by the terminal and basal plates. While their appearances vary among species, the basic components are conserved [10]. Though the exact protein composition of this region of the cilium is currently being elucidated [11], the transition zone is emerging as the key site for regulation of ciliary function. Transitional fibers are thought to be required not only for docking and anchoring the basal body to the membrane, but also as docking sites for IFT particles and associated molecular motors, both of which are destined for the ciliary compartment. It has been further proposed that the fibers form part of a pore complex (comparable to nuclear pores), regulating traffic into and out of the cilium [12]. Similar to these fibers, the Y-linkers and ciliary necklace are proposed to regulate trafficking into and out of the cilium [10,13]. Many of the proteins associated with cilia function appear to interact dynamically [14-16], and disruptions of these interactions could play a substantial role in the phenotypic variability among the various ciliopathies as well as among individuals with the same condition [17]. More specifically, interactions between proteins, which when defective cause ciliopathies such as Meckel syndrome, nephronophthisis (NPHP) and Joubert syndrome, appear to be required for basal body anchoring and establishment of a ciliary gateway [11,18]. Disruptions of these protein networks, particularly at the ciliary transition zone, result in disruption of ciliogenesis and also in defective signal transduction [11,19].

Sensory cilia are additionally specialized by cell type to transduce the specific function or modality of that particular cell. For example, olfactory cilia detect odorants via specific receptors on the ciliary membrane, the connecting cilia in mammalian photoreceptors continuously transport various proteins to support the outer segments, and sensory cilia lining the kidney tubules sense fluid flow through the nephron.

Initial identification of disease causing genes in ciliopathies such as Bardet-Biedl syndrome (BBS) or NPHP, suggested that each ciliopathy had a distinct set of disease-causing genes. However, in recent years it has become apparent that there is considerable overlap between ciliopathy genes, suggesting that phenotypic variability among ciliopathies is caused by a combination of the nature of the mutation and the contribution of other allelic variations. Mutations in genes such as *CEP290 *can cause nearly all ciliopathies [20]; others such as *BBS4 *or *NPHP5 *are restricted to two [21]. In general, genes causing BBS and Meckel syndrome overlap as do those causing Senior Loken syndrome and NPHP [11,18,21].

The theory that 9+2 cilia solely mediate motile functions, while the 9+0 cilia have a strictly sensory function is now considered an oversimplification. Non-motile olfactory sensory cilia have a 9+2 structure [22], while the 9+0 cilia at the embryonic node are motile and generate leftward flow, required for establishment of asymmetry [23,24]. Further, ependymal cilia in the brain ventricles are proposed to have a sensory function in addition to their well-characterized motile function [5]. Intriguingly, motile cilia on human airway epithelial cells were recently found to express bitter taste receptors. Activation of these receptors increases intracellular calcium levels, thus stimulating beat frequency and so initiating a defensive mechanism against noxious substances [25]. These examples would suggest that additional motile cilia with sensory function are probably awaiting discovery and that the simplistic classification of cilia will need to be reconsidered. In fact, cilia with a 9+3 microtubule triplet have also been identified [26], although the specific function of this configuration is still unknown.

As the structural basis of all cilia is a ring of nine microtubules, ciliopathies could alternatively be viewed as diseases of microtubule function, with the cilium representing a specialized extension of the cytoskeleton. Whereas microtubule defects inside the cell might be mitigated due to compensatory roles of other proteins, the highly specialized ciliary transport machinery may be particularly sensitive to perturbations in microtubule function/transport due to its remote location and lack of compensatory subunits. Many of the ciliopathy genes code for proteins that localize to the basal body and/or transition zone and, as such, may exert a regulatory role on ciliary function. As microtubules are highly abundant in this region, their association and interaction with ciliary proteins are essential for ciliary function. In addition, we must consider the possibility that proteins important for cilia-related functions may also be required for non-ciliary microtubule and cytoskeletal associated processes [27-29].

## Ciliary Proteins - Beyond the Cilium

If the cilium is regarded as a specialized extension of the cytoskeleton, it is not surprising that many ciliary proteins are also beginning to emerge as key players in other cellular processes that involve the cytoskeleton. These include cell cycle progression and vesicular transport, both of which are heavily dependent on the configuration of the microtubule architecture within a cell.

The basal body, which anchors the cilium, is one of the microtubule-organizing centers of the cell, and recent work has identified a crucial link between ciliogenesis and cell cycle progression (reviewed in Kim and Tsiokas [30]). In cells with a single primary cilium, the basal body is derived from the distal end of the mother centriole which becomes encapsulated by Golgi-derived vesicles in a directional manner [31]. Prior to ciliogenesis, the mother centriole docks at the plasma membrane [32]. Further fusion of additional vesicles enables the cilium to protrude away from the cell body and into the extracellular matrix. Hence, growth and maintenance of cilia function is heavily dependent not only on IFT but also on internal trafficking of components to the basal body, which also utilizes IFT proteins [33-36].

**Figure 2:**
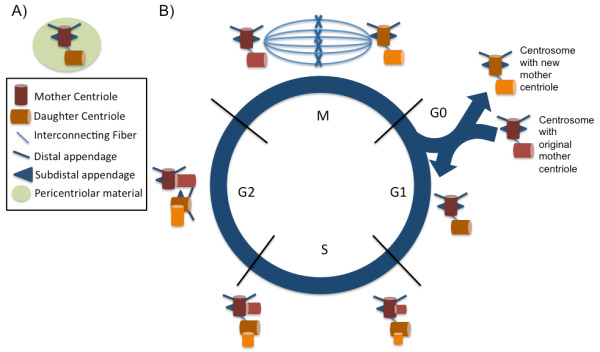
**Centrosome duplication during the cell cycle**. **(A) **Centrosomes are comprised of two centrioles (mother and daughter) connected via an interconnecting fiber. The mother centriole has additional distal and sub distal appendages. The centrioles are surrounded by a matrix of proteins, the pericentriolar material (PCM). **(B) **During the cell cycle, each centriole (the original mother and daughter centriole) duplicates once, growing a new daughter centriole from their sides. The original mother centriole duplicates at a faster rate than the original daughter centriole. The original daughter centriole acquires additional appendages and thus becomes a new mother centriole. Mitosis separates the two centrosomes (duplicated centrioles) resulting in two cells each with a differentially aged mother centriole. Differences between these cells regarding cell fate and regulation are beginning to emerge.

As the basal body derives from the mother centriole, the timing of ciliogenesis is closely related to mitosis, mitotic spindle deconstruction, and centriole duplication. Cilia customarily nucleate and grow during the G1-G0 phase of the cell cycle and then retract during mitosis [6]. The centrosome in G1 cells is comprised of two centrioles, termed the mother and daughter centriole. Each of the two centrioles in the centrosome self-duplicates once during the cell cycle, but their rate of maturation is asymmetric [37]. Upon self-duplication, both centrioles become mother centrioles with newly associated daughter centrioles. Consequently, the two centrosomes in a G2 cell have either the older (original) mother centriole or a newer mother centriole [38]. As the rate of maturation is asymmetric, this results in two differentially aged centrosomes that segregate upon cell division (Figure 2). Each new cell obtains a centrosome with either the older mother centriole or the younger mother centriole. Segregation of differentially aged mother centrioles affects subsequent cell fates and attributes, including the growth of a primary cilium [39]. Additional aspects of how centrioles and centrosomes transition to become basal bodies, which is thoroughly discussed in a recent review by Kobayashi and Dynlacht [40], involves proteins required for both centriole migration as well as cilia assembly and disassembly.

Asymmetric centrosomal inheritance has been implicated in subsequent cell fate decisions in *Drosophila *neuroblasts and male germline cells [41-43]. In mouse cortical neuroepithelium, inheritance of the mother centrosome correlated with a progenitor cell fate, whereas inheritance of the daughter centrosome was associated with differentiated progeny cells [43]. Asymmetric centrosomal inheritance after cell division also affects the timing of cilia growth [39]. Cells inheriting the older mother centrosomes appear to generate cilia sooner than those with younger daughter centrosomes. Differences in composition of both pericentriolar material (PCM) and pericentriolar satellites (sub-distal and distal appendages) between the two centrioles might contribute to the asymmetric rate of division and subsequent cell fate determination. The differential localization of various signaling proteins to the older *vs*. younger centriole of dividing sister cells adds a further layer of complexity to signaling regulation [39]. Asymmetric localization of proteins to dividing centrioles could also allow for potential establishment of apical-basal cell polarity within that cell.

These findings, namely that asymmetric distribution of proteins around the centriole is also important during both cell cycle progression and ciliogenesis, support the concept that the basal body region plays a critical regulatory role. Other contributors to such functions include the ciliary-related proteins CEP290, CP110, and Cep97, which are highly involved in cilia assembly and regulation and are also asymmetrically localized to the basal body region [13,44,45]. At a regulatory level, the centriolar kinesin Kif24 (see section on kinesins), which preferentially localizes to mother centrioles, has been found to prevent cilia assembly via recruiting and/or stabilizing CP110 at the distal end of mother centrioles [46]. Even in 'mature' basal bodies, asymmetric distribution of proteins such as Dishevelled have been shown to be required for cellular polarization [47].

In the emerging association of cilia and cell cycle progression, two dynein binding proteins, Ned1 and Tctex-1, have been shown to regulate cell cycle progression via effects on cilia length [48,49]. Both these proteins localize to the base of the cilium (Ned1 at the mother centriole and Tctex-1 at the transition zone) and are thought to control cilia length regulation, which in turn determines cell cycle progression. This observation could explain the presence of only a single primary cilium per cell during tissue morphogenesis and development when cells continue to divide. Once cells terminally differentiate, they are able to form multiple cilia, which is not possible prior to terminal differentiation as the single cilium must retract during cell division [6]. It would be interesting to know if the expression of Ned1 or Tctex-1 was down-regulated in differentiated, post-mitotic cells.

As IFT proteins have been shown to be required for intracellular trafficking to the cilium, not just along the cilium, it is plausible that these components might also be required for additional microtubule related transport. Intriguingly, the intraflagellar transport protein IFT88, required for ciliary anterograde transport, has also been shown to be required for spindle orientation during mitosis [29,50], thus coupling cell cycle progression, centriole division, and microtubule transport. As part of a dynein-1 driven complex, IFT88 appears to transport microtubule-nucleating proteins to the spindle poles, which are required for formation of astral microtubule arrays and subsequent correct spindle formation and orientation. This suggests possible roles for IFT proteins in cytoplasmic cellular mechanisms. Additionally, IFT20, another IFT-related protein, is known to facilitate transport of proteins from the Golgi to the base of the cilium [34]. IFT20 also interacts with IFT57 and IFT88 at the immune synapse in non-ciliated T-lymphocytes [28]. Similarly, in non-ciliated retinal neurons, IFT20, IFT57, and IFT52 have been shown to transport cargo vesicles to the dendritic post-synaptic terminal [36]. These non-ciliary localizations could however represent possible ciliary remnants formed in these cells. Additionally, it should be noted that the plasma membrane over the centrosome could be functionally equivalent to the ciliary membrane and so share similar protein composition.

While *in vitro *evidence is mounting that many ciliary proteins have additional, alternate roles away from the cilium, it is important to note that *in vivo *genetic evidence is as yet lacking. Many mouse and zebrafish mutants which lack cilia appear to survive well into development [51,52]. Nevertheless, this could reflect a potentially high degree of functional redundancy and/or developmental stage and tissue specificity. As will be discussed in subsequent sections, the distinction between cilia versus basal body proteins may also be a factor in further dissecting the roles for individual 'cilia' proteins. Primary cilia biology is still in its infancy, so it is not naïve to assume that we may find many more secondary/additional roles of ciliary proteins, which will greatly advance our understanding of ciliary function and general cell biology.

## Cilia in Wnt Signaling - the Importance of the Basal Body

In recent years, many different signaling pathways, such as canonical and non-canonical Wnt[7], Shh[53-57], FGF[58,59], Notch[60,61], mTor[62], PDGF[63], and Hippo[64] signaling, have been shown to be associated with the primary cilium. However, ciliary signaling and the cytoskeleton appear to play particularly important roles in the modulation of the Wnt pathways. Integral Wnt signaling is crucial in modulating the cytoskeleton and, reciprocally, Wnt signaling requires an intact cytoskeleton. Downstream effects of non-canonical Wnt signaling (also referred to as planar cell polarity [PCP]) result in cytoskeletal-actin rearrangements while components of the canonical Wnt signaling pathway associate heavily with microtubule and cytoskeletal components.

While both canonical and non-canonical Wnt signaling are activated by Wnt molecules binding to distinct membrane-bound receptors, downstream steps diverge. Canonical Wnt signaling results in stabilization of cytoplasmic β-catenin, which subsequently enters the nucleus and initiates transcription of Wnt target genes. In contrast, downstream events of PCP signaling result in changes in cell morphology (actin dynamics, cell polarity, cell shape, and cell change) rather than transcription. This process ultimately results in the directed orientation of cells relative to a planar axis within the epithelium. While the precise role of cilia in Wnt signaling is currently being dissected [7], a key step will be to distinguish the role of the basal body and transition zone, which may be independent of the cilium. Also of consequence will be the distinction between the role of ciliary proteins in the cilium versus in the cell body, where they may play additional downstream roles in signal transmission.

One of the first indications that cilia might be involved in Wnt signaling was the identification of two key PCP components, Inversin (Nephrocystin 2) and Dishevelled (Dvl), at the basal body (Table 1) [47,65,66]. Mutations in Inversin causes one of the ciliopathies NPHP as well as *situs inversus *[67]. This was followed by observations that knockdown of several BBS and other ciliary-associated genes (*Kif3a*, *Ift88*, *Ofd1*) resulted in hyperactive canonical Wnt responses [68-70]. Mutations in other ciliary-associated proteins also suggested a role for cilia in restraining Wnt signaling (Chibby, Seahorse, SREBP1c) [71-75]. These differences might be attributed to tissue- and time-dependent variables, but also to the varying localization and function of these proteins.

**Table 1:** Summary of proteins associated with canonical/non-canonical Wnt signaling and cilia

Canonical Wnt	Cilia or Wnt association	Reference
Wnt proteins		
Inv (NPHP2)	Ciliary localization and physical interaction with Dvl	[65,66]
Dvl	Docking and polarization of basal bodies, basal body localization, targeted for degradation by Inv	[47,66]
Cilia-associated proteins		
Bbs-associated	Hyperactive Wnt response in knockout cell lines	[68]
Kif3a^a^	Up regulation of cellular Wnt response in knockout cell lines and mutant mice	[68,69]
Ift88^a^	Up regulation of cellular Wnt response in mutant mice	[69,70]
Ift40	Increase in expression of canonical Wnt pathway genes in kidney of mutant mice	[91]
Ift20	Increase in nuclear beta-catenin and expression of Wnt target genes in kidney of mutant mice	[89]
Ofd1	Up regulation of cellular Wnt response in mutant mice	[69]
Chibby	Binds beta-catenin preventing nuclear entry negatively regulating Wnt signaling	[71-73]
Seahorse	Binds to Dvl, constrains Wnt signaling in zebrafish	[74]
SREBP1c	Over expression disrupts ciliogenesis and increases canonical Wnt signaling in Xenopus.	[75]
Ahi1/Jbn	Abrogated Wnt signaling in kidney and cerebellum of mutant mice, facilitates beta-catenin entry into nucleus	[19,106,143]
**Non-canonical Wnt**		
PCP effectors		
Inturned	Highly expressed in ciliated tissue, required for ciliogenesis. Actin assembly, Rho localization, docking of basal bodies	[100,144,47,147]
Fuzzy	Required for axoneme elongation, predicted role in vesicular trafficking	[100,144]
Fritz	Expressed in ciliated tissue, required for ciliogenesis, mutations identified in human ciliopathies	[101]
Core PCP proteins		
Dubroya	Regulates ciliogenesis, Apical Actin assembly	[148]
Frizzled	Defective ciliogenesis at zebrafish kupffers Vesicle	[148]
Dvl	Regulates ciliogenesis, Actin assembly, Rho localization, docking of basal bodies, associated with human ciliopathy proteins TMEM216 and TMEM67	[47,149]
Celsr2/Celsr3	Regulates ciliogenesis in multiciliated ependymal cells via basal body docking at apical plasma membrane	[150]
Prickle	Regulates cilium length in zebrafish	[151]
Vangl2^a^	Localizes to some cilia, xenopus basal body localization and ciliogenesis, zebrafish conflicting data	[82,83,96]

^a^Further described in the legend

The primary cilium was first implicated in suppression of canonical, β-catenin-dependent Wnt signaling, while being required for non-canonical Wnt signaling (PCP). One of the first studies to make this suggestion implicated inversin (Inv), a basal body protein associated with cystic kidney disease, as a molecular switch between the two Wnt signaling pathways. It was suggested that this occurred via targeting of cytoplasmic Dishevelled (Dvl) for degradation at the basal body. Another cilia-associated protein, the kinesin-like protein Kif3a, is thought to restrain canonical Wnt signaling by restricting the CK1-dependent phosphorylation of Dvl, which results in an active beta-catenin destruction complex, limiting beta-catenin induced transcription. It is possible that defects in protein interactions at the basal body, rather than in intraflagellar transport (IFT), could be responsible for dysregulation of Wnt signaling in cilia mutants. Such a suggestion is consistent with the findings that both IFT-mutant mice and mutant zebrafish do not show disruption of canonical Wnt signaling. ^a^*Axin 2 *(Wnt target gene) and transgenic Wnt reporter normal in *ift88*, *ift72*, and *kif3a *mouse embryos [51]. No Wnt phenotype in ift88 zebrafish [52]. Conflicting zebrafish vangl2 data could be due to varying animal mutants and analysis methods.

It is also important to note that there have been some studies that suggest there is no connection between cilia and Wnt signaling. Maternal-zygotic ift88 zebrafish mutants lack all cilia, yet display normal canonical and non-canonical Wnt signaling [52]. At midgestation *Ift88*, *Ift72*, and *Kif3a *mutant mouse embryos, all of which lack primary cilia, show normal expression of the canonical Wnt target gene, *Axin2*, and normal activation of a transgenic canonical Wnt reporter [51]. Mouse embryonic fibroblasts derived from these mutants also respond normally to Wnt ligands [51]. However, these findings do not exclude the possibility that there are tissue- and time-dependent differences in ciliary association with Wnt signaling. One explanation for the normal Wnt responsiveness shown by these zebrafish and mouse cilia mutants might be that the basal bodies and ciliary transition zones in these organisms are not disrupted. It is possible that if the cilium is missing but the basal body and surrounding structures remain, this allows some degree of Wnt signaling to continue. This strengthens the argument for the basal body or transition zone region being a key modulator of the Wnt response.

### Non-canonical Wnt (PCP) signaling

The PCP signaling pathway was initially described in *Drosophila *and includes polarized localization of core PCP proteins, which ultimately leads to the establishment of polarized cell morphology [76,77]. In vertebrates, PCP signaling has been shown to be required for convergent extension [78], defects in which result in disrupted neural tube closures, as well as misorientation of hair cells in the mammalian cochlea [79] and hair follicles in the epidermis [80]. One of the early indications that ciliary proteins may be linked to PCP regulation was the observation of PCP defects in ciliary mutant cochlea, linking basal body polarization with PCP regulation [81,82]. In recent years PCP signaling has also been found to be required for the orientation of basal bodies in several other tissues, including positioning of motile monocilia at the node, gastrocoel, and Kupffer's vesicle, which is involved in the establishment of left-right asymmetry in fish [47,83-86].

As discussed, initial observations of PCP defects in ciliary mutants (mice, zebrafish) [66,82] suggested a role for cilia in PCP signaling. Concurrently, the emerging role of PCP signaling in renal cyst development strengthened this hypothesis as most of the ciliopathies are associated with kidney diseases. Inversin has been suggested as a molecular switch between the canonical and non-canonical signaling pathways by targeting Dishevelled for degradation via the APC complex at the basal body [66].

In the mammalian kidney, cilia/basal body dysfunction has also been suggested to affect the orientation of mitotic spindles, associated with defective tubule division and resultant polycystic kidney disease [87]. Defective ciliogenesis, centrosomal amplification, centrosome positioning, and mitotic spindle orientation have been observed in the kidneys of centrosome/basal body mutants (MKS1, MKS3, IFT20) [88-90]. Interestingly, a more recent study in which IFT40, an IFT complex A protein (as opposed to IFT20 which is an IFT complex B protein), was deleted from mouse collecting ducts showed that deletion of IFT40 causes cystic kidneys without altering centrosome or mitotic spindle position [91]. Similarly, data from mice with mutations in polycystic kidney disease genes *Pkd1*, *Pkd2*, and *Pkhd1 *are conflicting and suggest that loss of oriented cell division alone does not initiate cyst formation. While oriented cell division is disrupted in *Pkhd1 *mutants, this alone appears not to be sufficient to produce kidney cysts. In contrast mutations in *Pkd1 *and *Pkd2 *do result in cyst formation despite normal oriented cell division, suggesting that loss of PCP-related oriented cell division is not sufficient to generate kidney cysts [92]. Therefore, while basal body positioning, or even earlier centrosome positioning, appears to require some degree of intact PCP regulation, it is unclear whether cilia or basal body integrity can affect PCP signaling. If so, how and what is required first; intact PCP for centrosome positioning or intact centrosomes for subsequent polarizing events?

Ciliary localization of several core PCP proteins adds a further layer of complexity to dissecting this relationship. The protocadherin Fat4 was shown to localize to the cilium, and *Fat4^-/- ^*mice display severe PCP phenotypes thought to be caused by abnormal orientation of mitotic spindles [93]. Two other core PCP proteins, Vangl2 and Dvl, have also been reported to associate with the primary cilium. Whereas Dvl, which is degraded by inversin, docks basal bodies to the apical membrane preceding ciliogenesis [94], Vangl2 is responsible for asymmetric positioning of motile cilia [95]. Vangl2 has been shown to localize along the ciliary axoneme in olfactory neurons [82] and in cilia of multiciliated ependymal cells [96]. In addition, Vangl2 has also been shown to interact biochemically and genetically with the ciliary protein BBS8 in zebrafish, resulting in laterality defects due to defective fluid flow at the Kupffer's vesicle, the equivalent of the embryonic mouse node [97]. Fluid flow influences centriolar movement and contributes to orientation of motile cilia in conjunction with PCP [96-98].

It is possible that correct basal body and centrosome positioning could be required for subsequent regulation of cytoskeletal architecture. The basal body, as a MTOC, could regulate the nucleation and outgrowth of microtubules; indeed, several of the BBS proteins have been shown to participate in microtubule nucleation and growth [27,99]. Evidence for cilia and PCP regulation of the actin cytoskeleton is emerging. Many PCP proteins are involved in Rho-mediated apical actin assembly, Rho localization, and basal body docking, which includes tethering of membrane bound vesicles (Table 1). *Xenopus *and *Drosophila *PCP effectors Inturned and Fuzzy are responsible for ciliogenesis and have been shown to affect apical actin assembly and microtubule polarity but not nucleation. Defects may occur in cytoskeletal assembly that impair movement of basal bodies and centrioles [100]. The PCP component Fritz controls localization of the cytoskeletal protein Septin, crucial for collective cell movement and ciliogenesis in *Xenopus *embryos. A recent study found an enrichment of non-synonymous coding changes in human *Fritz *in two ciliopathies: Bardet-Biedl and Meckel-Gruber syndrome [101]. Of note, the identification of the polycystic kidney disease protein PC1 at cell adherens junctions and desmosomes, and not just cilia and basal bodies, indicates a possible additional role for PC1 in control of cytoskeletal rearrangement potentially down stream of PCP signaling [102].

As will be discussed in subsequent sections, regulation of various signaling molecules (including Rho A and Dvl) could occur at the basal body. Examples of this include the Smurf ubiquitin ligases, which target proteins for degradation by proteasomes. These proteins regulate PCP signaling via localized Prickle degradation by the Par6-Dvl2-Smurf complex [103]. In *Smurf *mutant cochlea, polarity proteins (Prickle 1) are not asymmetrically degraded at the region of the basal body as in wild-type animals. This further implicates the basal body as a regional signaling center assisting the activation and/or degradation of signaling proteins. A recent proteomic screen found several PCP-associated proteins in the human centrosome such as PRICKLE3, SCRIB, CCDC66, and Albatross [104]. This screen also identified asymmetrically localizing centriolar proteins and differentially aged centrioles in cell division (Figure 2), which appear to be important in cell polarization and stem cell fate determination via asynchronous microtubule spindle orientation and primary cilia growth [39,104].

Similar to the previous quandary regarding PCP and centrosome/basal body positioning, a key question that remains to be elucidated is which comes first: is intact PCP signaling required for ciliogenesis, or is an intact cilium necessary for correct PCP signaling? As downstream effects of PCP signaling are associated with actin and microtubules, one could envisage a possible feedback loop where intact cytoskeletal architecture and function is in turn necessary for cilia formation or function. Another key question is whether the cilium itself is responsible for the defects observed in some PCP mutants. Many of the cilia-related proteins are now being identified in other cellular locations and could possibly be required for additional intracellular functions. As suggested before, the cilium is heavily dependent on ciliary proteins, and defects in their function may be more readily observable at this organelle.

### Canonical Wnt signaling

**Figure 3:**
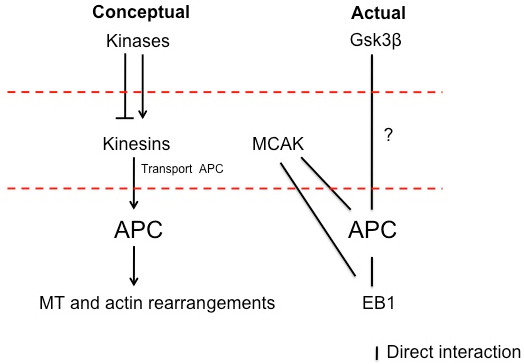
**Diagrammatic overview of Wnt involvement with the cytoskeleton**. Transport of APC by kinesin motors plays an important role in microtubule stabilization, activation of protein kinases and cell polarization, which could all be regulated via the Gsk3β kinase. Phosphorylation of kinesins via kinases controls their sub-cellular localization and activity. Gsk3β is one of the central kinases predominantly associated with Wnt signaling and may influence many functions of kinesins through regulating cargo binding. Phospohrylation of APC by Gsk3β decreases the interaction of APC with microtubules, subsequently decreasing microtubule stability. Gsk3β could be considered a master regulator of kinesin control over MT dynamics, due to its ability to regulate a range of kinesins.

At first glance, the β-catenin-dependent canonical Wnt signaling pathway appears relatively straightforward. Wnt ligands bind and activate the Frizzled class of receptors, thereby stabilizing β-catenin, which then enters the nucleus and subsequently activates Wnt target genes [105]. This pathway requires a large number of regulatory components, which delicately balance multiple feedback mechanisms. In the absence of Wnt, β-catenin is sequestered and degraded by a degradation complex consisting of Axin, Adenomatosis polyposis (APC), Glycogen synthase 3 beta (Gsk3β), and Casein kinase 1 (CK1). Upon Wnt stimulation, this degradation complex translocates to the membrane where it is inactivated via phosphorylation, subsequently stabilizing β-catenin and releasing it for entry into the nucleus. *Jouberin*, mutations of which cause the ciliopathy Joubert syndrome, has been shown to facilitate β-catenin translocation to the nucleus [106]. As will be further discussed, it is possible that the basal body is the site of regulation of the APC complex, localization of which could in turn affect its regulatory activity. Many components of canonical Wnt signaling localize or associate with the cilium (Table 1, Figure 3). Similar to the question as to what regulates what with regard to cilia and PCP signaling, is the dependence of canonical Wnt signaling on cytoskeletal architecture. As discussed below, cilia/basal body associations between APC and canonical Wnt-related kinesins are beginning to emerge. These findings further enhance our understanding of the role of the cilium/basal body in Wnt regulation or vice versa.

As we further discuss the various components required for Wnt signaling and their association with cilia and basal bodies, one factor to consider is which is more dependent on the other: is intact cytoskeletal architecture (including cilia and basal bodies) required for Wnt signaling, or is Wnt signaling required for the establishment of a fully functional cytoskeleton encompassing a cilium and basal body?

### APC

The tumor suppressor protein APC is one of the key components in the canonical Wnt signaling pathway. Most of the scientific literature is focused around its ability to bind and induce degradation of β-catenin. However, there are additional roles for this protein involving the organization of cytoskeletal networks [107]. APC has been identified at a number of intracellular sites (plasma membrane, cytoplasm, centrosomes, kinetochores, nucleus) [108], as well as at the distal ends of microtubules at the edges of migrating epithelial cells [109-111]. APC associates with the plasma membrane in an actin-dependent manner and regulates actin cytoskeletal networks required for cell polarization and directional migration (reviewed in Akiyama and Kawasaki [107]). The APC protein also binds and stabilizes microtubule subsets, associating both directly and indirectly with microtubules via its C-terminal domain. Direct interaction with the microtubule binding protein EB1 [112] enhances microtubule assembly and stabilization [113], while transport of APC towards microtubule plus-ends involves several kinesins (kinesin-1, KIF3 and KIF17, kinesin-2) [109,114]. Transport of APC to its various cellular locations by kinesin motors plays a vital role in microtubule stabilization, activation of protein kinases, and cell polarization and therefore kinesins are of paramount importance to these processes. Importantly, in regard to ciliogenesis, APC has been shown to be one of the few proteins that localizes asymmetrically to mother *vs*. daughter centrioles and may play a role in primary cilia formation and function [104]. This most likely occurs via regulation of actin or microtubules at the basal body region. Only a small number of proteins display this distinctive feature of asymmetrical centriole distribution in a cell cycle-dependent manner; other known examples include microtubule-related proteins such as EB1, which interacts directly with APC, as well as cilia-related proteins such as CP110 [104]. The EB proteins, EB1 and EB3 (but not EB2), also directly promote cilia biogenesis via stabilizing minus-end microtubules at the centrosome and basal body [115]. Interestingly, lack of EB1 and EB3 results in defective microtubule anchoring at the centrosome/basal body and vesicular trafficking at the base of the cilium. The authors suggest that EB1 and/or EB3 disrupt ciliogenesis through effects at the basal body or cilium, not through alterations of cytoplasmic microtubule plus-end dynamics. Both EB1 and EB3 interact with proteins implicated in centrosomal microtubule minus-end anchoring and ciliary vesicle transport such as PCM1, Ninein, Cep290, Cep170 [115]. Studies in both mammalian cells and *Chlamydomonas reinhardtii *have identified EB1 localizing to subdistal appendages of the mother centriole/basal body [116,117], which are involved in microtubule minus-end anchoring [115,118].

As the centrosome and basal body region at the base of the cilium is beginning to emerge as a cellular signaling centre, it is not difficult to imagine that APC, in its role as part of the β-catenin destruction complex, might be anchored to this region. In support of this theory, other related components are also being identified in this area such as members of the kinesin class of motor proteins.

### Kinesins

The kinesins, a class of motor protein, exert a regulatory effect on Wnt signaling. Many proteins that bind to microtubules participate in the regulation of microtubule dynamics. These include the kinesins, which are powered by the hydrolysis of ATP. Although kinesins share similar motor domains, they exhibit a variety of functions, including regulation of microtubule dynamics. Kinesins are known to be crucial for ciliogenesis and intraflagellar transport [15,119]. Recently, a centriolar kinesin Kif24 was shown to remodel a cellular subset of microtubules regulating ciliary assembly. Kif24 preferentially localizes to the mother centriole and is required for centrosomal localization of CP110 [46]. N-kinesins are commonly associated with anterograde transport, while C-kinesins are involved with retrograde transport. M-kinesins, by contrast, do not possess a motor function, but play a role in microtubule stabilization [120]. The ability of kinesins to control microtubule dynamics enables these molecules to exert regulatory function. Such regulatory mechanisms are coupled to various signaling pathways, which could, in turn, control their associations with microtubules or regulate microtubules and/or microtubule dynamics directly. As kinesins can be both signaling targets and signaling intermediates, kinesin control over microtubule dynamics is complex.

Wnt signaling at the centrosome is one of the ways in which kinesins exert regulatory functions. Not only are kinesins required for localization of APC to the centrioles and centrosome, but another kinesin, MCAK (also referred to as KIF2C), also associates predominantly with these structures. As a member of the kinesin-13 family (other members of which include KIF2A and KIF2B), MCAK has been shown to be critical for microtubule dynamics and organization [120]. This promiscuous kinesin, localizes dynamically to centrosomes, kinetochores, the spindle mid-zone during cell division, and to microtubule tips [121-123]. MCAK dimers bind to microtubule ends (plus and minus) and are able to induce a conformational change causing the tubulin to bend and subsequently destabilize. As MCAK has been shown to associate directly with APC^99^, MCAK dysfunction could hinder correct localization of APC, thereby disrupting Wnt signaling.

MCAK has also been shown to associate with the microtubule-associated protein EB1 via its C-terminus at growing ends of microtubules [124,125]. When associated with EB1, MCAK appears to be in an inactive conformation. This could provide a mechanism to facilitate fast switches between growth and depolymerization of microtubules, which is of particular importance during ciliogenesis. As EB1 also associates directly with APC and plays an important role in microtubule stabilization, it is possible that these three molecules are involved in a regulatory feedback loop (Figure 3).

In summary, kinesins are not only required for trafficking of structural or signaling components to and along the cilium, but are also required for stabilization and regulation of the cytoskeletal infrastructure along which these molecules travel. In these capacities the kinesins are able to exert significant control over various cellular mechanisms.

### Factors that regulate both canonical and non-canonical Wnt signaling

Identification of ciliary proteins that affect both canonical and non-canonical Wnt signaling are not surprising, considering that the cilium can potentially be seen as a branch point bisecting the two pathways. These include a family of proteins, the GTPases. The GTPases are structurally related to kinesins, however, they hydrolyze GTP instead of ATP. GTPases are involved with cellular trafficking, and several members of the ARF-like family of small GTPases have been implicated in ciliary function [126,127]. As this subset of GTPases is generally associated with membrane trafficking and microtubule-associated processes, it is most likely that they are required for the transition between post-Golgi vesicular trafficking to the basal body and kinesin-dependent IFT within the cilium, at the transition zone.

**Figure 4:**
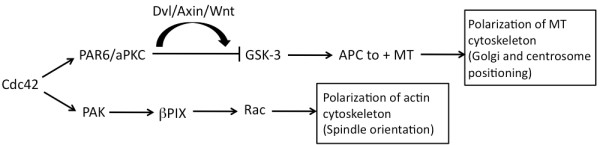
**Diagram of Cdc42 regulation of cytoskeletal rearrangement**. Cdc42 can control polarity via two separate pathways. Activation of Par6/aPKC inhibits GSK-3, which results in polarization of microtubules (MT). Rac-dependent polarization of the actin cytoskeleton is due to Pak activated βPIX.

Cdc42, a Rho family GTPase, is a key player in Wnt-dependent cytoskeletal rearrangements and a key regulator of cell polarity. Cdc42 acts as a molecular switch, modulating a wide range of signaling pathways. In cellular trafficking, Cdc42 functions at several steps of endocytosis, recycling, and biosynthesis, influencing polarized trafficking in epithelial cells [128]. In its role as a polarity regulator, Cdc42 has been shown to orchestrate polarity of microtubule and actin cytoskeletons via two different signal transduction pathways [129] (Figure 4). First, direct interaction with Par6 leads to activation of aPKC and subsequent accumulation of APC at the plus-end tips of microtubules promoting polarization of the microtubule cytoskeleton. The requirement of Dvl, Axin, and the Wnt 5a ligand in this process implicates the Cdc42/Par6 complex in establishing microtubule polarity via transcription-independent non-canonical Wnt signaling [130]. Second, Cdc42 association with PAK results in activation of the Rac guanine nucleotide exchange factor βPIX and subsequent downstream Rac-dependent polarization of the actin cytoskeleton [129,131]. It has been suggested that the Par polarity complex can also affect trafficking, further linking the dual actions of Cdc42 [132]. Dvl has also been shown to directly regulate aPKC stabilization and activation promoting axon differentiation mediated by the Par6 complex in cultured hippocampal neurons [133].

Importantly, the Cdc42/Par6/aPKC complex has been localized to the primary cilium and has been shown to be necessary for ciliogenesis in mammalian cells [134,135]. What does this mean for cilia? As several other components (for example, Dvl, kinases, GTPases) required for subsequent signaling events also localize near the cilium (basal body), it is possible that Cdc42 is functioning under the control of a potential basal body 'signaling center'. Cdc42 can be regarded as a molecular switch, and its ciliary localization puts it at the putative site of regulation between canonical and non-canonical signaling at the base of the cilium. A more comprehensive role for Cdc42 at the cilium could encompass vesicle transport and docking (fusion with the plasma membrane) in the delivery of proteins necessary for ciliogenesis. Such a view is supported by recent findings that Cdc42 localizes the exocyst (a highly conserved eight-protein membrane trafficking complex), to the base of the cilium, where it is possibly stabilized by binding to the Par complex via Par6 [136]. Once the exocyst complex is stabilized, it can then target and dock further cilia-bound vesicles. A more upstream role for Cdc42 involvement in ciliogenesis could involve basal body positioning, centrosome regulation, spindle orientation, and cellular polarity, all via the ability of Cdc42 to regulate the cytoskeleton (Figure 4).

Cdc42 is not the only GTPase associated with the cilium. Other GTPases that also localize to the cilium could function in a similar way to, or in conjunction with, Cdc42. One such GTPase is ARL6/BBS3, one of the BBS proteins not associated with the core group of BBS proteins comprising the IFT-participating 'BBSome', a stable protein complex which functions in primary cilia biogenesis [14]. This suggests a more distinct function for ARL6/BBS3, which may be required for regulating membrane trafficking at the base of the cilium and consequently affecting cilia assembly and subsequent ciliary signaling events [137]. BBS3 localizes to distal ends of the basal bodies akin to transition fibers where the ciliary-bound vesicles dock and fuse at the base of the cilium. An additional BBS protein, BBS1 (another component of the BBSome), is known to interact with Rabin8, a GDP-GTP nucleotide exchange factor (GEF) for the small GTPase RAB8 [14]. Rabin8 co-localizes with the BBSome at the basal body while RAB8 enters the cilium in its GTP-bound state and is necessary for ciliogenesis. In turn RAB8 interacts with Rabaptin5, which also localizes to the basal body and is associated with the IFT protein Ey11/DYF11. It is thought that this trio (RAB8/Rabaptin5/DYF11) may be critical for the transition between vesicular trafficking to the ciliary membrane and IFT. Furthermore in *C. elegans*, the small GTPase RAB5 has been shown to localize to the base of the cilium and regulate polycystin 1 and 2 removal from the cilium [138].

Other proteins that appear to intercept the two signaling pathways include pVHL. The pVHL protein has been proposed to control ciliogenesis by orienting microtubule growth. This protein is a cargo of Kif3 and has been shown to interact with the polarizing PAR3-PAR6-aPKC complex, suggesting that these proteins may operate in the same pathway to regulate cortical growth of microtubules and formation of cilia [139]. Precisely how pVHL signals via kinesin motors or microtubules to control cellular processes such as polarity as well as ciliogenesis remains to be investigated. Intriguingly, KIF17 has recently been shown to stabilize microtubules and contribute to establishing epithelial cell polarity, purportedly by contributing to APC localization at microtubule plus ends [114]. Another aspect to consider is the association of both kinesins and IFT proteins with mitotic spindle assembly [29]. It is thought that while all of the kinesin-13s are required for spindle assembly, it is likely that various members might contribute to spindle polarity and dynamics through different pathways.

Identification of molecules that are already known to function as molecular switches between signaling pathways, suggests that there are possibly more awaiting discovery. These may also have the capacity to control or 'fine tune' Wnt signaling decisions. The basal body, an emerging signaling 'hotspot' perfectly located at the base of the 'sensory-loaded' cilium might be an ideal cellular location for the switch.

## Conclusions

We are only beginning to understand the role of the cilium and its biological influence. The cilium is more than just a microtubular appendage anchored to the cell via the basal body. The multiple parts of the cilium from the basal body to the distal tip (Figure 1) are just beginning to be defined. The cilium may serve as a 'receptive device' for signals in the cell's environment, helping to regulate development and other cell functions. One key question that remains to be addressed is whether the cilium could potentially release signaling factors for communication with its environment.

The base of the cilium is the most crucial part of this 'communication device', as the incoming signals need to be ordered and regulated. It is perhaps not a coincidence that the basal body doubles as the MTOC of the cell. The basal body is most likely regulating other internal signaling and trafficking events as well. Regulation of this region is a hugely critical and as yet underexplored area. However, many kinases, phosphatases, kinesins, and proteosomal proteins that localize to this region are being identified. Of particular consideration in regard to cilia, two of the kinases known to affect Wnt signaling, CKID and PPPIC, have been identified in the ciliary proteome [68].

The base of the cilium is not just a signaling center, but also a sorting center. An emerging concept is that of scaffold proteins acting as hubs for controlling the flow of cellular information (spatial and temporal organization of molecules) within a cell [140]. Several basal body proteins could be seen as such potential scaffold proteins. For example, Cep290 is an enormous protein and appears to have wide-ranging effects on both basal body/ciliary structure and regulation [141]. This concept fits with the notion of a ciliary pore akin to the nuclear pore, regulating ciliary protein composition [7]. Is this gate-keeping function restricted to ciliary proteins? Might the basal body be regulating other trafficking in the cell? As an MTOC for the entire cell, the basal body could possibly regulate much more than just what is happening at the cilium. Perhaps it can be compared to a bus terminal for cilia-related proteins: where cargo is loaded and unloaded prior to being sent along its way and 'bus routes' can be regulated and established. Importantly, a recent paper demonstrated that Wnt signaling regulates spindle asymmetry to generate asymmetric distribution of β-catenin in *C. elegans *[142]. This is one of the first examples of nuclear protein localization being controlled via microtubules and suggests that microtubule organization may have direct impact on gene expression in the nucleus. It also suggests that Wnt signaling may be both up- and downstream of microtubule modulation.

The concept of the cilium as a potential regulator (molecular switch) between canonical and non-canonical Wnt signaling has been suggested [66] and could explain why there is conflicting data on the association (whether positive or negative) between cilia and Wnt signaling. The interplay between regulation of Wnt signaling and the basal body/MTOC could expand the mechanisms by which cilia are involved in the molecular switch between canonical and non-canonical signaling, as all of these molecular pathways converge at the basal body. It is possible that defects in protein interactions at the basal body, rather than in IFT, could be responsible for dysregulation of Wnt signaling in cilia mutants.

It would certainly be an oversimplification to assume that a single type of cilium would suffice for the needs of every cell. The key to our understanding of cilia function will be to distinguish the characteristics of cilia in differing cells and tissues. The recent explosion of ciliary research is likely to continue for the foreseeable future as this long forgotten organelle reveals its inner secrets.

## Abbreviations

APC: adenomatosis polyposis; CK1: Casein kinase 1; BBS: Bardet-Biedl syndrome; GSK3β: glycogen synthase 3b; IFT: intraflagellar transport; NPHP: nephronophthisis; PCM: pericentriolar material; PCP: planar cell polarity; Shh: Sonic hedgehog.

## Competing interests

The authors declare that they have no competing interests.

## Authors' contributions

HMS prepared the manuscript, MK edited the manuscript. Both authors read and approved the final manuscript.
